# Long-Pentraxin 3 Affects Primary Cilium in Zebrafish Embryo and Cancer Cells via the FGF System

**DOI:** 10.3390/cancers12071756

**Published:** 2020-07-01

**Authors:** Jessica Guerra, Paola Chiodelli, Chiara Tobia, Claudia Gerri, Marco Presta

**Affiliations:** 1 Department of Molecular and Translational Medicine, University of Brescia, 25123 Brescia, Italy; j.guerra@unibs.it (J.G.); paola.chiodelli@unibs.it (P.C.); chiara.tobia@unibs.it (C.T.); claudia.gerri@crick.ac.uk (C.G.); 2 Francis Crick Institute, London NW1 1AT, UK; 3 Italian Consortium for Biotechnology (CIB), 25123 Brescia, Italy

**Keywords:** FGF, long-pentraxin 3, primary cilium, cancer, zebrafish

## Abstract

Primary cilium drives the left-right asymmetry process during embryonic development. Moreover, its dysregulation contributes to cancer progression by affecting various signaling pathways. The fibroblast growth factor (FGF)/FGF receptor (FGFR) system modulates primary cilium length and plays a pivotal role in embryogenesis and tumor growth. Here, we investigated the impact of the natural FGF trap long-pentraxin 3 (*PTX3*) on the determination of primary cilium extension in zebrafish embryo and cancer cells. The results demonstrate that down modulation of the *PTX3* orthologue *ptx3b* causes the shortening of primary cilium in zebrafish embryo in a FGF-dependent manner, leading to defects in the left-right asymmetry determination. Conversely, *PTX3* upregulation causes the elongation of primary cilium in FGF-dependent cancer cells. Previous observations have identified the PTX3-derived small molecule NSC12 as an orally available FGF trap with anticancer effects on FGF-dependent tumors. In keeping with the non-redundant role of the FGF/FGR system in primary cilium length determination, NSC12 induces the elongation of primary cilium in FGF-dependent tumor cells, thus acting as a ciliogenic anticancer molecule in vitro and in vivo. Together, these findings demonstrate the ability of the natural FGF trap PTX3 to exert a modulatory effect on primary cilium in embryonic development and cancer. Moreover, they set the basis for the design of novel ciliogenic drugs with potential implications for the therapy of FGF-dependent tumors.

## 1. Introduction

Primary cilia are antenna-like organelles protruding from most mammalian cells [1] to act as chemosensors and mechanosensors for external stimuli [2]. The primary cilium is composed by an axoneme made by microtubule triplets anchored at the cell by a basal body [3]. The basal body derives from the mother centriole of the centrosome [4], which is fundamental to cell division. The cilium is resorbed prior to mitosis to release the centrioles. Then, ciliogenesis occurs shortly after cytokinesis has been completed [3].

Recently, the zebrafish embryo has been used as a model for the study of primary cilia functions in development and diseases [5,6]. During embryonic development, primary cilia in the embryonic node are involved in the left-right asymmetry process [7] and genetic defects in primary cilia are associated with a variety of pathological conditions that are grouped under the name “ciliopathies” (reviewed in [8,9,10]). Due to their role in the modulation of various signaling pathways, including Hedgehog (Hh) and Wnt, dysregulation of primary cilia plays an important role also in cancer progression [11,12,13]. Thus, “ciliotherapy” approaches have been proposed for cancer therapy in which ciliogenic drugs hamper tumor cell proliferation in part through induction of the primary cilium [14].

Long-pentraxin 3 (*PTX3*) is a soluble patter recognition receptor involved in the innate immunity arm [15]. PTX3 plays a role in wound healing/tissue remodeling, cardiovascular diseases, fertility, and infectious diseases [16]. In common with other pentraxins, the *C*-terminal portion of PTX3 includes the pentraxin-signature [17] whereas its unique *N*-terminal extension is responsible for binding to different members of the fibroblast growth factor (FGF) superfamily (including FGF2, FGF6, FGF8b, FGF10, and FGF17), thus preventing their interaction with all members of the FGFR family (FGFR1–4) [18,19,20]. Thus, PTX3 inhibits FGF-dependent responses, such as endothelial cell proliferation in vitro and angiogenesis in vivo [19,21,22], and exerts an oncosuppressive effect on FGF-dependent tumors, including multiple myeloma, melanoma, fibrosarcoma, lung, prostate, and bladder cancers [23,24,25,26,27,28,29]. In a therapeutic perspective, these findings led to the identification of the PTX3-derived small molecule NSC12 as the first orally available FGF trap able to inhibit the activity of all the members of the canonical FGF family by preventing their binding to FGFR1, 2, 3, and 4 [24]. This confers to NSC12 the capacity to inhibit the tumorigenic, angiogenic, and metastatic activity of tumors in which ligand-dependent activation of the FGF/FGF receptor (FGFR) system represents a driving force [24].

The FGF/FGFR system modulates primary cilium length in human and murine fibroblasts and chondrocytes [30]. In addition, it controls primary cilium extension in zebrafish embryos [31]. However, despite its ability to act as a natural FGF trap, no data are available about a possible involvement of PTX3 in primary cilium length determination under physiological and pathological conditions, including cancer. In the present research, we investigated the effect of the modulation of *PTX3* expression on primary cilium extension in zebrafish embryo and cancer cells. Our data demonstrate that down modulation of the *PTX3* orthologue *ptx3b* causes the shortening of primary cilia in zebrafish embryo in a FGF-dependent manner, leading to defects in the left-right asymmetry determination. Conversely, human *PTX3* (*hPTX3*) upregulation causes the elongation of primary cilia in different FGF-dependent cancer cell lines, including TRAMP-C2 prostate cancer cells that originate from the transgenic adenocarcinoma of the mouse prostate (TRAMP) model [32]. Accordingly, the PTX3-derived FGF trap NSC12 acts in vitro and in vivo as a ciliogenic anticancer molecule on FGF-dependent tumor cells. Together, our findings demonstrate for the first time the ability of PTX3 to exert a modulatory effect on primary cilium, shedding a new light on the manifold biological functions of this soluble pattern recognition receptor in embryonic development and cancer. In addition, they set the basis for the design of novel PTX3-derived ciliogenic drugs able to affect a different aspect of the biology of FGF-dependent tumors.

## 2. Results

### 2.1. In Silico Analysis of hPTX3 Co-Orthologs in Zebrafish

According to the Gene and HomoloGene databases at NCBI [PMID: 25398906], two putative co-orthologs of *hPTX3*, named *ptx3a* and *ptx3b*, are present in the zebrafish genome. They are located on the chromosomes 18 and 2, respectively, and are organized in three exons and two introns as their human counterpart.

CLUSTAL Omega alignment (https://www.ebi.ac.uk/Tools/msa/clustalo/) of the FASTA protein sequences of *hPTX3* (NP_002843.2), zPtx3a (XP_021329017.1), and zPtx3b (XP_694358.3) showed that *hPTX3* shares 39.74% amino acid sequence identity with Ptx3a and 41.13% identity with Ptx3b (Figure S1). Moreover, the canonical pentraxin signature and the conserved cysteine residues Cys-210 and Cys-271 are present in both zebrafish co-orthologs. Based on the Synteny Database program (http://syntenydb.uoregon.edu/synteny_db/), both zebrafish genes share a syntenic cluster of genes with *hPTX3*. In detail, when considering a window site of 50 genes, *ptx3a* shows three conserved genes (*selt1b*, *veph1*, *ccnL*1) (Figure S2A) whereas *ptx3b* shows ten conserved genes (*ccnl1b*, *golim4b*, *pccD10*, *slitrk3*, *samd7*, *sec62*, *gpr160*, *skil*, *phc3*, *prkc*l) (Figure S2B). Together, in silico data indicate that *ptx3a* and *ptx3b* are two bona-fide co-orthologs of *hPTX3*. In this research, we focused our attention on *ptx3b* due to its higher amino acid identity and conserved synteny with *hPTX3*.

### 2.2. Temporal and Spatial Expression of ptx3b during Zebrafish Development

**Figure 1:**
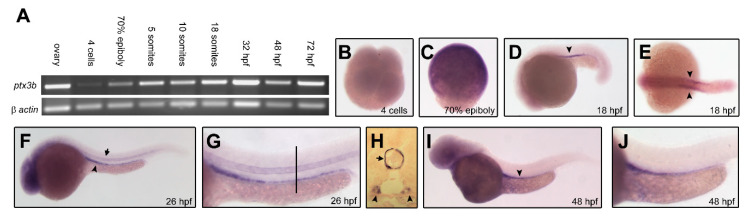
Zebrafish down modulation of the long-pentraxin 3 orthologue (*ptx3b*) expression. (**A**) RT-PCR analysis of *ptx3b* expression in the ovary and at the indicated developmental stages; (**B**–**J**) whole-mount in situ hybridization (WISH) analysis of *ptx3b* expression in zebrafish embryo at the indicated developmental stages; (**B**,**E**) dorsal view; (**C**,**D**,**F**,**G**,**I**,**J**) lateral view; (**G**,**J**) high magnification of the trunk region of embryos in (**F**) and (**I**); respectively; (**H**) transverse cross section of the trunk region of a 26 h post-fertilization (pf) embryo at the level of the black bar in (**G**); arrowheads in (**D**–**F**,**H**,**I**) indicate the pronephric ducts; arrows in (**F**) and (**G**) indicate the notochord.

The expression of *ptx3b* was analyzed at different stages of zebrafish embryo development by RT-PCR and whole-mount in situ hybridization (WISH). As shown in Figure 1A, *ptx3b* expression, detectable in the ovary, is absent at the four-cell stage, increases during epiboly, and remains constant from the five-somite stage (ss) to the 72 h post-fertilization (hpf) stage. During somitogenesis, the expression of *ptx3b* is restricted to the pronephric duct primordia where it was observed up to 48 hpf (Figure 1D–J). In addition, *ptx3b* is expressed in a transient manner at 26 hpf also in the notochord (Figure 1F,G), as highlighted by the analysis of paraffin-embedded transverse cross sections of the embryo trunk (Figure 1H), to be lost at 48 hpf (Figure 1I,J).

### 2.3. ptx3b Knockdown Causes Defects in the Determination of Left-Right Asymmetry in Zebrafish

**Figure 2:**
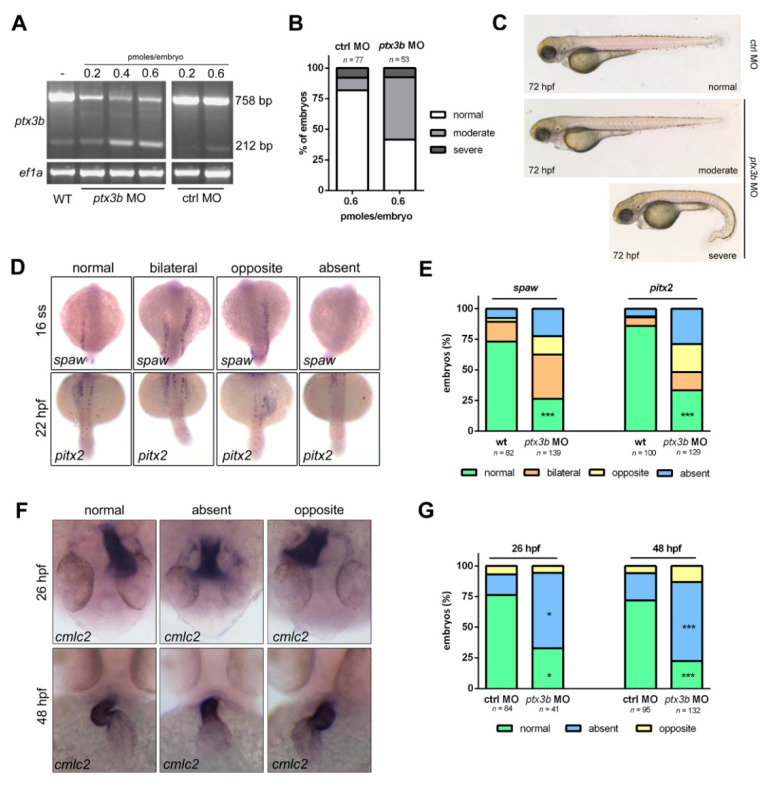
*ptx3b* downregulation causes left-right asymmetry defects in zebrafish embryo. (**A**) RT-PCR analysis showing the effect of different doses of ctrl or *ptx3b* morpholino (MO) on *ptx3b* expression in 28 hpf injected embryos. The efficacy of *ptx3b* MO is demonstrated by the presence of a specific 212 bp band in *ptx3b* MO-injected embryos, which confirms the occurrence of exon skipping. *ef1a* serves as control; (**B**) percentage of embryos showing normal, moderate, or severe phenotype at 72 hpf after the injection of 0.6 pmoles of ctrl MO or *ptx3b* MO, respectively; (**C**) representative bright field whole mount pictures of the phenotypes observed in ctrl and *ptx3b* morphants; (**D**) WISH representative pictures of the alterations of the expression of the laterality genes *spaw* and *pitx2* observed in *ptx3b* morphants at 16 ss and 22 hpf, respectively; (**E**) percentage of embryos with normal or altered expression of *spaw* and *pitx2* in WT (untreated) and *ptx3b* MO-injected embryos. Data are the mean of 4 and 3 independent experiment, respectively; (**F**) WISH representative pictures of *cmlc2* expression during normal and altered cardiac jogging and looping at 26 and 48 hpf, respectively; (**G**) percentage of embryos with normal or altered cardiac jogging and looping in ctrl MO and *ptx3b* MO-injected embryos. Data are the mean of 2 and 3 independent experiments, respectively. * *p* < 0.05, *** *p* < 0.001, ANOVA. *n*, total number of analyzed embryos.

In order to assess the role of *ptx3b* on zebrafish embryo development, we used an antisense morpholino (MO) knockdown approach. To this purpose, a splicing MO (*ptx3b* MO) was designed to target the exon 2/intron 2 border of the *ptx3b* transcript. A five-mismatch nucleotide MO, unable to bind the *ptx3b* mRNA (ctrl MO), was used as control. As shown in Figure 2A, RT-PCR analysis performed at 28 hpf confirmed the targeting efficacy of the *ptx3b* MO that caused the skipping of exon 2 in the *ptx3b* transcript of embryo morphants when compared to controls. Based on these results, the dose of 0.6 pmol/embryo was considered as the optimal dose of *ptx3b* MO to be used for further studies.

When compared to controls, 40% of *ptx3b* morphants showed no morphologic alterations and 50% of them exhibited only moderate defects (Figure 2B,C). Given the hypothesis that the FGF trapping activity of *PTX3* may result in primary cilium alterations and consequent defects in the left-right asymmetry process [33], WISH analysis was performed to investigate the expression of laterality genes and the positioning of visceral organs in control and *ptx3b* morphants. As shown in Figure 2D,E, the majority of 16-ss and 22 hpf *ptx3b* morphants showed bilateral, right-sided, or absent expression of *spaw* and *pitx2* transcripts, two Nodal-related genes normally expressed at the left side of the embryo [34].

Next, we examined the positioning of heart, liver, and pancreas in control and *ptx3b* MO-injected embryos by WISH analysis using the tissue-specific probes *cmlc2*, *prox1*, and *islet1*, respectively. As shown in Figure 2F,G, the *cmlc2^+^* cardiac jogging and looping processes, which occur at 26 and 48 hpf, respectively, were absent in the majority of *ptx3b* morphants. Moreover, *ptx3b* down-modulation caused significant alterations in the positioning of *islet1^+^* dorsal pancreatic bud and of *prox1^+^* liver when assessed at 24 hpf and 48 hpf, respectively (Figure S3).

### 2.4. ptx3b Modulates Primary Cilium Length Determination via the FGF/FGFR System in Zebrafish

**Figure 3:**
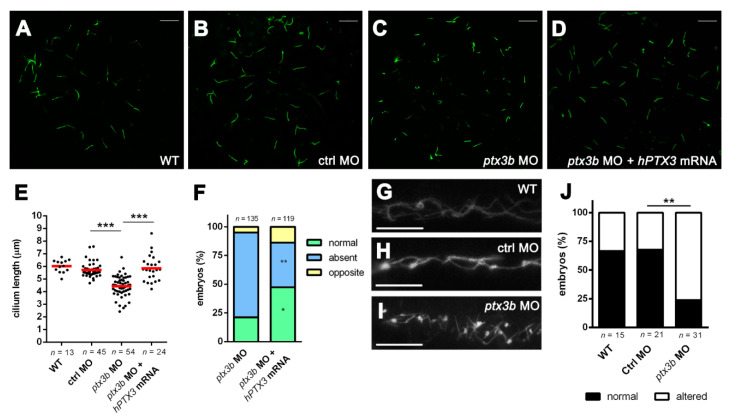
*ptx3b* knockdown alters primary cilium in zebrafish embryo. (**A**–**D**) Representative images of acetylated α-tubulin^+^ ciliary axonemes in the Kupffer’s vesicle (KV) of 10 ss WT (untreated) embryos (**A**) and of embryos injected with ctrl MO (**B**); *ptx3b* MO (**C**); and *ptx3b* MO plus human *PTX3* (*hPTX3*) mRNA (**D**); scale bar: 10 µm; (**E**) primary cilium length (in µm) in the KVs of untreated embryos (WT) and of embryos injected with ctrl MO, *ptx3b* MO or *ptx3b* MO plus *hPTX3* mRNA (500 pg/embryo). Each dot represents the mean length of primary cilia measured in a single KV. *n*, total number of examined KVs. Data were obtained in three independent experiments, *** *p* < 0.001, Student’s *t*-test; (**F**) percentage of embryos showing *cmcl*^2+^ normal or altered cardiac looping at 48 hpf after injection with *ptx3b* MO or *ptx3b* MO plus *hPTX3* mRNA. Data are the mean of three independent experiments. * *p* < 0.05, ** *p* < 0.01, ANOVA. *n*, total number of examined embryos; (**G**–**I**) representative pictures of acetylated α-tubulin^+^ ciliary axonemes in pronephric ducts of WT, ctrl MO, and *ptx3b* MO-injected embryos at 48 hpf stage. Scale bar: 10 µm; (**J**) percentage of embryos with normal or altered primary cilia organization in pronephric ducts. ** *p* < 0.01, Z-test, two-tailed.

Primary cilia of the embryonic node, named Kupffer’s vesicle (KV) in zebrafish [5], are involved in the determination of the left-right asymmetry during development [7,35]. On this basis, the length of KV primary cilia was measured in control and *ptx3b* morphants after acetylated α-tubulin immunostaining [6]. As shown in Figure 3, primary cilia of the KV are significantly shorter in *ptx3b* morphants when compared to control animals. At variance, no difference in the number of KV primary cilia was observed in *ptx3b* MO-injected embryos versus controls (mean ± S.E.M. equal to 21.5 ± 2.7 (*n* = 30) versus 22.7 ± 2.0 (*n* = 35) cilia per KV, respectively). To confirm the specificity of the *ptx3b* MO effects, one-cell stage embryos were co-injected with the *ptx3b* MO and an excess of *hPTX3* mRNA. As shown in Figure 3D,E, *hPTX3* mRNA was able to rescue the shortening of KV primary cilia caused by the *ptx3b* MO. Accordingly, rescued embryos showed a reduced percentage of cardiac looping defects when compared to *ptx3b* morphants (Figure 3F).

Notably, in keeping with the alterations observed in the KV, *ptx3b* morphants showed also structural defects of the primary cilia of the pronephric ducts [36] (Figure 3G–J).

**Figure 4:**
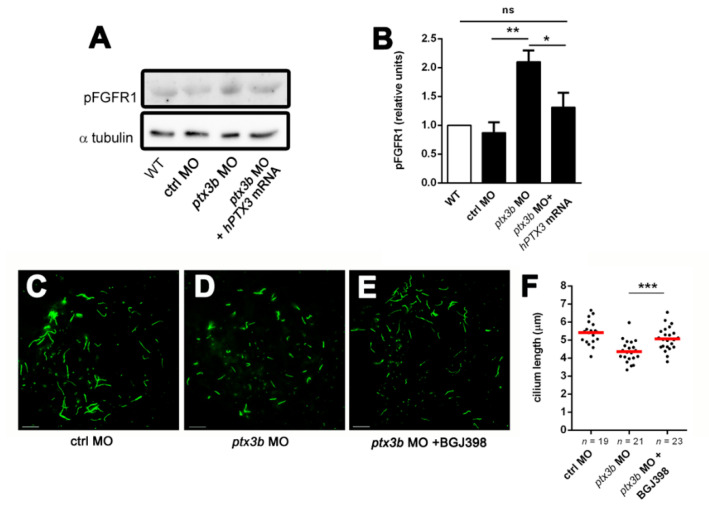
*ptx3b* regulates primary cilium length in zebrafish via the fibroblast growth factor (FGF)/FGF receptor (FGFR) system. (**A**) Western blot analysis of protein extracts from WT (untreated), ctrl MO, *ptx3b* MO, and *ptx3b* MO plus *hPTX3* mRNA-injected embryos probed with an anti-pFGFR1 antibody; uniform loading of the gel was confirmed using an anti-α tubulin antibody; (**B**) densitometric analysis of pFGFR1 levels normalized to α-tubulin expression. Data are the mean ± S.E.M. of 3 independent experiments, ** *p* < 0.01, * *p* < 0.05, Student’s *t*-test; (**C**–**E**) representative images of the acetylated α-tubulin^+^ KV ciliary axoneme of 10 ss embryos injected with ctrl MO or *ptx3b* MO and of *ptx3b* MO-injected embryos treated at shield stage with BGJ398 (50 nM). Scale bar: 10 µm; (**F**) primary cilium length (in µm) in the KVs of embryos treated as in (**C**–**E**). Each dot is the mean length of primary cilia measured in a single KV. *n*, total number of examined KVs. Data are from three independent experiments, *** *p* < 0.001, Student’s *t*-test.

Given the capacity of *PTX3* to bind different members of the FGF family [29], these data raise the hypothesis that a modulation of the activity of the FGF/FGFR system might be responsible for the shortening of primary cilia in *ptx3b* morphants. Indeed, *ptx3b* MO injection caused a significant increase of FGFR1 phosphorylation in zebrafish embryos that was fully abolished by the co-injection with an excess of *hPTX3* mRNA (Figure 4A,B). To substantiate this hypothesis, *ptx3b* MO-injected embryos were treated at the shield stage with the selective tyrosine kinase FGFR inhibitor BGJ398 (50 nM in fish water). As anticipated, BGJ398 treatment was able to rescue the length of KV primary cilia in *ptx3b* morphants (Figure 4C–F).

Together, these data show for the first time that *ptx3b* regulates the length of primary cilium axoneme during zebrafish embryo development by modulating the activity of the FGF/FGFR system, thus playing a non-redundant role in the left-right asymmetry process and visceral organ positioning in zebrafish.

### 2.5. PTX3 Regulates Primary Cilium Length in Cancer Cells via the FGF/FGFR System

Alterations of primary cilia may contribute to cancer progression [11,12]. Based on the results obtained in *ptx3b* zebrafish morphants and the well-known impact of *PTX3* on FGF-dependent tumors [23,24,25,26,27,37], we decided to investigate whether the modulation of *PTX3* expression may affect primary cilia in FGF-dependent cancer cells.

**Figure 5:**
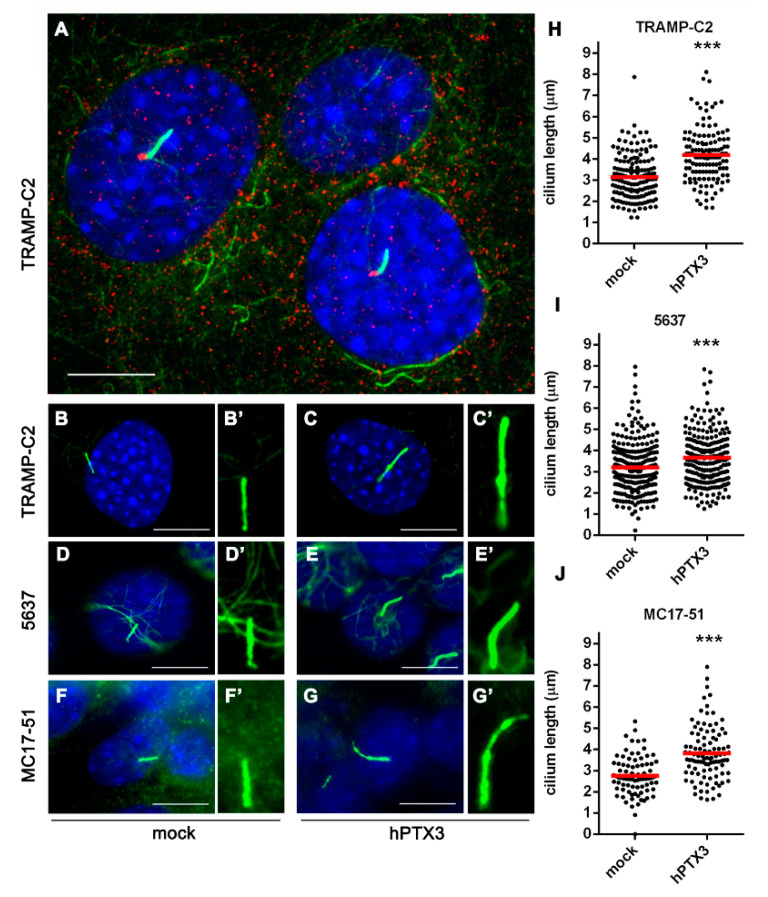
*PTX3* overexpression increases primary cilium length in different tumor cell lines. (**A**) Representative image of murine prostate TRAMP-C2 cells double immunostained with anti-acetylated α-tubulin (green) and anti-γ-tubulin (red) antibodies to visualize primary cilium axoneme and basal body, respectively. Nuclei were counterstained with DAPI (blue); (**B**–**G’**) representative images of primary cilium axoneme in mock (**B**,**B’**) and *hPTX3* overexpressing (**C**,**C’**); TRAMP-C2 cells; mock (**D**,**D’**) and *hPTX3* overexpressing (**E**,**E’**) human bladder carcinoma *hPTX3*-5637 cells; mock (**F**,**F’**) and *hPTX3* overexpressing (**G**,**G’**) murine fibrosarcoma MC17-51 cells. Scale bar: 10 µm; (**H**–**J**) the length (µm) of acetylated α-tubulin^+^ cilia was measured in mock and *hPTX3* overexpressing cells using the ImageJ software. Black dots represent individual cilia; red bars show the mean value. Data are from three independent experiments, *** *p* < 0.001, Student’s *t*-test.

Stemming from the observation that primary cilium number and length decrease in a subset of pre-invasive human prostatic lesions [13], we measured the length of primary cilia in TRAMP-C2 prostate cancer cells and in their *hPTX3*-overexpressing counterpart (*hPTX3*-TRAMP-C2 cells), characterized by a reduced FGFR signaling and tumorigenic potential [37]. As anticipated, *hPTX3* overexpression resulted in a significant elongation of primary cilium in *hPTX3*-TRAMP-C2 cells when compared to TRAMP-C2 cells (Figure 5 and Figure S4). Similar results were obtained in *hPTX3* transfectants that originated from FGF-dependent human bladder cancer 5637 cells [27] and murine fibrosarcoma MC17-51 cells [26] (Figure 5). At variance, *hPTX3* overexpression did not affect the percentage of ciliated tumor cells in all the cell lines tested (Figure S5).

**Figure 6:**
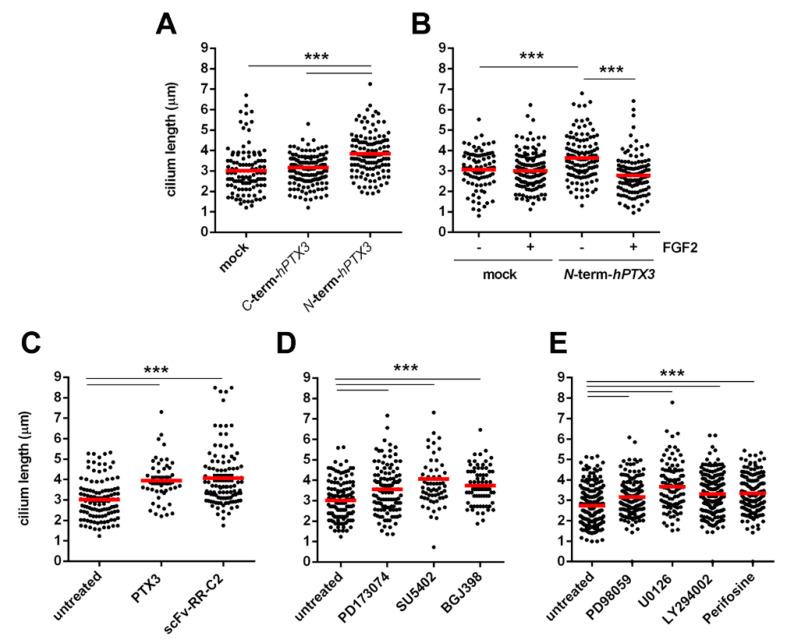
Inhibition of FGF signaling increases the length of primary cilium in TRAMP-C2 cells. (**A**–**E**) Cilia were visualized in serum-starved cells by acetylated α-tubulin immunostaining and their length was measured using the ImageJ software. Black dots represent individual cilia; red bars show the mean values. Data were obtained from three independent experiments, *** *p* < 0.001, Student’s *t*-test; (**A**) primary cilium length in TRAMP-C2 cells overexpressing the *C*-terminal or the *N*-terminal fragment of human PTX3; (**B**) primary cilium length in mock_TRAMP-C2 and *N*-term-*hPTX3*-TRAMP-C2 cells treated for 48 h with 30 ng/mL FGF2; (**C**) primary cilium length in TRAMP-C2 cells treated for 48 h with recombinant *PTX3* protein (66 nM) or with anti-FGFR1 single-chain antibody fragment scFv-RR-C2 (300 nM); (**D**) primary cilium length in TRAMP-C2 cells treated for 48 h with the tyrosine kinase FGFR inhibitors PD173074 (100 nM), SU5402 (100 nM), or BGJ398 (100 nM); (**E**) primary cilium length in TRAMP-C2 treated for 48 h with the MAPK inhibitors PD98059 (10 µM) or U0126 (1.0 µM) or with the PI3K inhibitors LY294002 (10 µM) or perifosine (1.0 µM).

PTX3 binds FGFs via its *N*-terminal domain [20]. To confirm the hypothesis that PTX3 may affect primary cilium length via the FGF/FGFR system in cancer cells, we measured the length of primary cilium in TRAMP-C2 cells stably transfected with the *N*-terminal *hPTX3* cDNA (*N*-term-*hPTX3*-TRAMP-C2 cells) or with the *C*-terminal *hPTX3* cDNA (*C*-term-*hPTX3*-TRAMP-C2 cells) [37]. As anticipated, overexpression of the FGF-binding *N*-terminal fragment of PTX3 resulted in an increase of the length of primary cilium when compared to mock-transfected cells, whereas overexpression of the PTX3 *C*-terminus was ineffective (Figure 6A). These results were confirmed by a rescue experiment in which treatment with exogenous recombinant FGF2 protein caused the shortening of the primary cilium in *N*-term-*hPTX3*-TRAMP-C2 cells with no effect on mock-TRAMP-C2 cells (Figure 6B).

In keeping with these observations, treatment with recombinant PTX3 protein or with the neutralizing anti-FGFR1 single-chain antibody fragment scFv-RR-C2 [38] resulted in the elongation of primary cilium in TRAMP-C2 cells (Figure 6C). Accordingly, treatment with the tyrosine kinase FGFR inhibitors PD173074, SU5402 or BGJ398, or with different inhibitors of FGFR downstream signaling pathways, including the MAPK inhibitors PD98059 and U0126 and the PI3K inhibitors LY294002 and perifosine, were able to extend the length of primary cilium in TRAMP-C2 cells (Figure 6D,E). Together, the results indicate that the blockade of the FGF/FGFR system by extracellular or intracellular inhibitors exerts a significant impact on the primary cilium in TRAMP-C2 cells.

**Figure 7:**
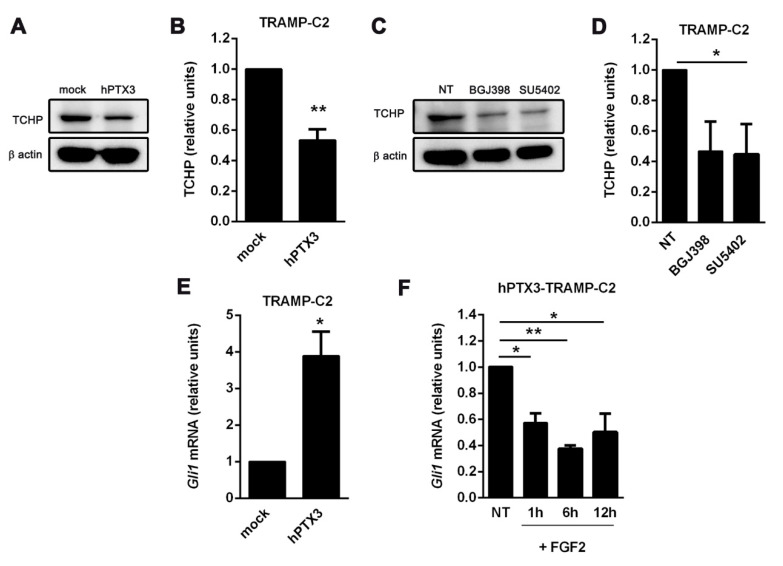
Inhibition of the FGF/FGFR system affects cilium-related signaling. (**A**) Serum-starved mock and *hPTX3*-TRAMP-C2 cell protein extracts were probed with an anti-trichoplein (TCHP) antibody. Uniform loading of the gel was assessed by probing the membrane with an anti-β actin antibody; (**B**) densitometric analysis of trichoplein levels normalized to β actin; (**C**) serum-starved mock-TRAMP-C2 cells were treated with the FGFR inhibitors BGJ398 (100 nM) or SU5402 (100 nM) for 48 h. After lysis, the extracts were probed with an anti-trichoplein antibody; (**D**) densitometric analysis of trichoplein levels normalized to β-actin; (**E**) *Gli1* expression in serum-starved mock and *hPTX3*_TRAMP-C2 cells; (**F**) serum-starved *hPTX3*-TRAMP-C2 cells were incubated with recombinant FGF2 (30 ng/mL) for 1, 6, or 12 h. Then, *Gli1* expression was evaluated by qRT-PCR and normalized to *Gaphd* mRNA levels. All data are the mean ± S.E.M. of 3–4 independent experiment, * *p* < 0.05, ** *p* < 0.01, Student’s *t*-test.

Activation of different tyrosine kinase receptors suppresses ciliogenesis in retinal epithelial cells by stabilizing the trichoplein-Aurora A complex following phosphorylation of the deubiquitinase USP8 [39]. Accordingly, the elongation of primary cilium observed in *hPTX3*-TRAMP-C2 cells was paralleled by a significant reduction of the intracellular levels of trichoplein (Figure 7A,B). Similar results were obtained in TRAMP-C2 cells treated with the tyrosine kinase FGFR inhibitors BGJ398 and SU5402 (Figure 7C,D).

Sustained activation of the FGF/FGFR system may affect primary cilium-dependent Hh signaling following the shortening of primary cilia [30]. On this basis, we investigated the impact of PTX3 on the activation of the Hh pathway in TRAMP-C2 cells using the downregulation of the expression of the Hh target transcriptional regulator GLI1 as a readout [40,41]. As shown in Figure 7E, *hPTX3* overexpression results in a significant increase of the levels of the *Gli1* transcript in *hPTX3*-TRAMP-C2 cells that was abolished by stimulation with exogenous FGF2 (Figure 7F).

### 2.6. The PTX3-Derived FGF Trap NSC12 Modulates Primary Cilium Length in Cancer Cells

**Figure 8:**
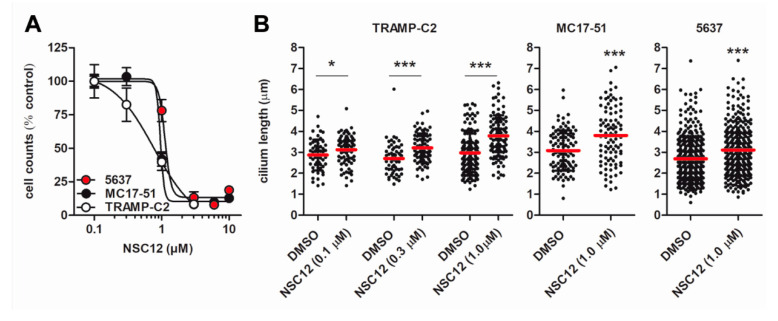
Ciliogenic activity of the FGF trap NSC12. Serum-starved TRAMP-C2, 5637, and MC17-51 cells were treated with the indicated concentrations of NSC12 or with the corresponding volume of vehicle (DMSO). After 48 h, cells were counted (**A**) and cilia lengths were evaluated with ImageJ software (**B**). Data are from two independent experiments, * *p* < 0.05, *** *p* < 0.001, Student’s *t*-test.

Previous observations from our laboratory led to the discovery of NSC12 as the first PTX3-derived small molecule endowed with a potent anti-FGF activity [24,42]. Orally available, NSC12 inhibits the growth, angiogenic potential, and metastatic activity of various FGF-dependent tumors with potential implications in cancer therapy [24]. On this basis, we evaluated the effect of NSC12 on cell proliferation and primary cilium length in FGF-dependent TRAMP-C2, 5637, and MC17-51 cells. As shown in Figure 8A and Figure S6, NSC12 inhibits the proliferation of FGF-dependent tumor cells with an ID_50_ equal approximately to 1.0 µM. Accordingly, when administered at 1.0 µM concentration, NSC12 causes a significant elongation of the primary cilium in all the cell lines tested (Figure 8B).

**Figure 9:**
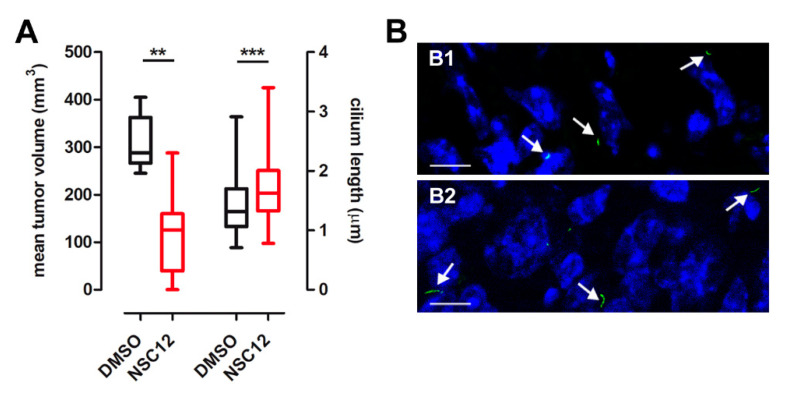
The FGF trap NSC12 exerts a ciliogenic activity on TRAMP-C2 tumors. (**A**) TRAMP-C2 cells were injected into the flank of C57BL/6 mice (10–15 animals/group). At tumor take, animals were treated i.p with NSC12 or vehicle. After 17 days, tumors were measured, harvested and frozen tissue sections were immunostained with an anti-acetylated α-tubulin antibody. The length of tumor cilia was measured in 40 microscopic fields from 3–5 tumors/group. Data are represented by box and whisker plots where the boxes extend from the 25th to the 75th percentiles, lines indicate the median values, and whiskers indicate the range of values. ** *p* < 0.01, *** *p* < 0.001, Student’s *t*-test; (**B**) representative images of TRAMP-C2 tumors treated with vehicle (**a**) or NSC12 (**b**) and immunostained with anti-acetylated α-tubulin antibody (green) to visualize primary cilia (arrows). Nuclei were counterstained with DAPI (blue). Scale bar: 10 µm.

Next, the capacity of NSC12 to exert a ciliogenic effect was evaluated in vivo on TRAMP-C2 tumor grafts. To this purpose, TRAMP-C2 cells were injected subcutaneously (s.c.) in syngeneic mice. At tumor take (30 days post-implantation), animals were treated i.p three times a week with NSC12 (5.0 mg/kg body wt) or vehicle. After 17 days, tumors were measured with calipers and harvested. Then, OCT embedded samples were immunostained with anti-acetylated α-tubulin antibody. In keeping with previous observations [24], NSC12 causes a significant inhibition of TRAMP-C2 tumor growth that was paralleled by a significant increase of the length of acetylated α-tubulin^+^ tumor cilium (Figure 9). At variance, NSC12 did not cause any change in α-tubulin immunoreactivity in TRAMP-C2 grafts (Figure S7).

## 3. Discussion

The primary cilium is a microtubule-based structure that protrudes from the surface of most mammalian cells. It functions as a cellular antenna that captures environmental signals and serves as a hub for developmental and homeostatic signaling pathways [43].

PTX3 is a soluble patter recognition receptor and a key player of the innate immunity arm with non-redundant functions in pathogen recognition and inflammatory responses [15,16]. In addition, PTX3 is endowed with the capacity to exert an antitumor activity by acting as a natural FGF trap [29]. In this research, we provide experimental evidence that *ptx3b*, an ortholog of *hPTX3*, affects zebrafish development by regulating the length of the axoneme of the primary cilia of the KV, playing a non-redundant role in the left-right asymmetry process and visceral organ positioning during zebrafish embryogenesis. These effects appear to be mediated by the capacity of Ptx3b to modulate FGF/FGFR signaling in zebrafish embryo, its downregulation leading to increased FGFR1 phosphorylation. This was paralleled by shortening of the primary cilium axoneme in the KV, rescued by treatment with the selective tyrosine kinase FGFR inhibitor BGJ398. These data extend recent observations about the capacity of FGFR1 or FGF ligand inactivation to cause primary cilium shortening in zebrafish and Xenopus embryos [31]. Accordingly, FGF signaling regulates the length of primary cilia in various stem, embryonic, and differentiated mammalian cell types [30]. In addition, human skeletal dysplasia caused by activating FGFR3 mutations are characterized by alterations of the primary cilium [30,44].

Compelling experimental evidence indicates that alterations of primary cilium may play an important role also in tumors by affecting various intracellular signal transduction pathways (reviewed in [8,12]). Moreover, alterations of primary cilium affect different aspects of tumor biology, including cancer cell autophagy and apoptosis, response to hypoxia, epithelial–mesenchymal transition, and drug resistance [45]. In this frame, various types of cancer cells fail to express the primary cilium, including pancreatic, breast, prostate cancer, and melanoma cells [13,45,46,47,48,49,50]. Hence, restoration of the primary cilium in cancer cells may provide novel opportunities for therapeutic antineoplastic interventions. In this frame, drug repurposing “ciliotherapy” approaches have been proposed to hamper tumor growth by induction of the primary cilium [14].

Deregulation of the FGF/FGFR network occurs in tumors due to gene amplification, activating mutations and oncogenic fusions [51,52,53]. The multifaceted FGFR signaling network is engaged in the progression of different FGF-dependent tumors by acting on both tumor and stromal cell compartments, thus affecting oncogenesis through different mechanisms, including cell-signaling deregulation, angiogenesis, and resistance to cancer therapies [54]. On this basis, different classes of therapeutics have been developed, including non-selective and selective tyrosine kinase FGFR inhibitors, monoclonal antibodies, and FGF ligand traps [55,56]. Some of them are under investigation in clinical trials in different FGFR-related cancer settings [53].

In this frame, *PTX3* has been shown to act as an oncosuppressor in different FGF-dependent tumors [24] by inhibiting tumor growth, neovascularization, and metastatic dissemination [29]. Here, we demonstrate that PTX3 has a ciliogenic effect on different cancer cell types. Experimental evidences indicate that this capacity is due to its FGF trap activity. (i) Overexpression in TRAMP-C2 cells of the FGF binding *N*-terminal fragment of PTX3 resulted in the elongation of primary cilium, whereas overexpression of the *PTX3 C*-terminus was ineffective; (ii) tyrosine kinase FGFR inhibitors and inhibitors of FGFR downstream signaling pathways cause primary cilium elongation in TRAMP-C2 cells. In parallel, tyrosine kinase FGFR inhibitors cause a significant reduction of the intracellular levels of trichoplein that, in complex with Aurora A, exerts suppressive effects on ciliogenesis [39]; (iii) *hPTX3* overexpression leads to *Gli1* upregulation, a marker of cilium-dependent Hh signaling [40], which is prevented by treatment with exogenous FGF2 protein.

NSC12 is a PTX3-derived small molecule able to bind all the extracellular members of the FGF family [24]. Numerous experimental evidences indicate that NSC12 may represent a prototype for the development of novel orally available therapeutic agents targeting FGF-dependent tumors [53]. Indeed, when compared to tyrosine kinase FGFR inhibitors, NSC12 is characterized by a reduced toxicity that may result in a more favorable therapeutic window [54]. Here we show that NSC12 inhibits the proliferation of FGF-dependent murine prostate cancer TRAMP-C2 cells, human bladder carcinoma 5637 cells, and murine fibrosarcoma MC17-51 cells (see also [26,27,37]) and that this inhibition is paralleled by the elongation of the primary cilium in all the cell lines tested. These data were confirmed in vivo, where NSC12 was able to inhibit tumor growth, and to induce a ciliogenic effect in TRAMP-C2 tumor grafts.

The present research focused mainly on the effect on primary cilium of FGF2 and FGFR1, the prototypic members of the FGF/FGFR system. Thus, further experiments will be required to assess the impact exerted on primary cilium by the modulation of different FGFs and FGFRs. Nevertheless, given the capacity of NSC12 to inhibit the activity of all the canonical FGFs and their interaction with all FGFRs [24], this small molecule FGF trap may represent the basis for the design of novel ciliogenic anticancer drugs able to affect different aspect of the biology of FGF-dependent tumors.

## 4. Materials and Methods

### 4.1. Chemicals

FGF2 was obtained from Peprotech (Rocky hill, NJ, USA), NSC12 was synthesized by M. Mor (University of Parma, Parma, Italy) as described [22], ScFv RR-C2 was isolated as described [42], *rhPTX3* was kindly provided by B. Bottazzi, (Humanitas Institute, Milan, Italy). PD173074, SU5402, PD98059, and LY294002 were from Sigma-Aldrich (St. Louis, MO, USA), BGJ393 was from Selleckchem (Houston, TX, USA), UO126 was from MedChem Express, (Monmouth Junction, NJ, USA) and perifosine was from Aeterna Zentaris (Frankfurt, Germany).

### 4.2. Bioinformatic Analysis

Zebrafish genomic sequences were analyzed using the Gene and HomoloGene databases at the National Center for Biotechnology Information (NCBI, https://www.ncbi.nlm.nih.gov/) (PMID: 25398906). The protein sequences of the two putative orthologue Ptx3a (XP_021329017.1) and Ptx3b (XP_694358.3) were aligned to the human *PTX3* sequence found on the NCBI database (NP_002843.2) with the program CLUSTAL Omega (https://www.ebi.ac.uk/Tools/msa/clustalo/), while syntenic regions were analyzed with the Synteny Database program (http://syntenydb.uoregon.edu/synteny_db/).

### 4.3. Zebrafish Maintenance and Collection

The wild-type zebrafish AB line was maintained at 28 °C under standard conditions and embryos were staged as described [57]. To examine embryos older than 22 hpf, fish water was added with 0.2 mM 1-phenil-2-thiourea (PTU, Sigma-Aldrich). For the observation of the in vivo phenotypes, embryos were anesthetized using 0.16 mg/mL Tricaine (Sigma).

### 4.4. Morpholino Injection

*ptx3b* MO (5′-CTGAATCATGTACCTGAGGGCAGAT-3′; Gene Tools, Philomath, OR, USA) targeting the exon 2/intron 2 border of the *ptx3b* transcript was injected at the indicated concentrations in 1–4 cell stage embryos. A five-mismatch nucleotide ctrl MO (5′-CTCAATGATCTACCTCACGGCAGAT-3′) was used as control. To confirm the targeting efficacy of the *ptx3b* MO, alternative splicing pattern analysis was performed on zebrafish embryos using appropriate primers (Table 1).

### 4.5. Whole-Mount In Situ Hybridization

Digoxigenin-labelled RNA probes were transcribed from linear cDNA constructs (Roche Applied Science, Penzberg, Germany). WISH was performed on embryos fixed in 4% PFA as described [58]. For sectioning, zebrafish embryos were post-fixed in 4% PFA after WISH, dehydrated in ethanol series, cleared in xilol, and paraffin-embedded overnight.

### 4.6. RT-PCR and qPCR Analysis

Total RNA was isolated from untreated and MO-injected embryos at different stages of development using TRIzol^®^ Reagent (Invitrogen, Carlsbad, CA, USA) according to the manufacturer’s instructions. Two micrograms of total RNA were retrotranscribed and 100 ng of cDNA were used for the evaluation of the alternative *ptx3b* splicing pattern by semi-quantitative RT-PCR analysis. The whole gels are shown in Figure S4.

For the evaluation of *Gli1* expression in cancer cells, total RNA was isolated with the same procedure. Then, 1/10th of the retrotranscribed cDNA was used for quantitative qPCR that was performed with the ViiA 7 Real-Time PCR System (Thermo Fisher Scientific, Waltham, MA, USA) using iTaq Syber Green Supermix (Biorad, Hercules, CA, USA) according to the manufacturer’s instructions. Samples were analyzed in triplicate and normalized with respect to the levels of *Gapdh* expression using the appropriate primers (Table 1).

### 4.7. Cell Cultures

Murine prostate adenocarcinoma TRAMP-C2 cells (ATCC CRL-2731) and *hPTX3* transfectants [37] were maintained in DMEM supplemented with 10% FBS, penicillin/streptomycin (100 U/mL and 10 mg/mL, respectively), 10 mM HEPES buffer, 0.5 mM 2-mercaptoethanol, 2.0 mM glutamine, 5 mg/mL bovine insulin (Sigma), and 10 nM DHT. Human bladder carcinoma 5637 cells (ATCC HTB-9) and murine fibrosarcoma MC17-51 cells (MC-TGS17-51) and their corresponding *hPTX3* transfectants were grown as described [26,27]. All cells were maintained in a humidified 5% CO2 incubator at 37 °C. Cells were maintained at low passage, returning to original frozen stocks every 3–4 months, and tested regularly for Mycoplasma negativity.

### 4.8. Cell Proliferation Assay

Cells were seeded on 48-well plates at 8 × 10^3^ cells/cm^2^. After 24 h, cells were treated with increasing concentrations of NSC12 in serum free DMEM (TRAMP-C2 and MC17-51 cells) or RPMI (5637 cells). After a further 48 h incubation, cells were trypsinized and viable cell counting was performed with the MACSQuant^®^ Analyzer (Miltenyi Biotec, Bergisch Gladbach, Germany).

### 4.9. Tumor Grafts in Mice

Animal studies were approved by the local animal ethics committee (Organismo Preposto al Benessere degli Animali, Università degli Studi di Brescia, Brescia, Italy) and by the Italian Ministero della Salute. All the procedures and animal care were conformed to institutional guidelines that comply with national and international laws and policies (EEC Council Directive 86/609, OJL 358, 12 December 1987).

C57BL/6 (Charles River, Calco, Italy) male mice were maintained under standard housing conditions. TRAMP-C2 cells (5 × 10^6^ in 200 µL of PBS) were injected s.c. into the dorsolateral flank of nine week-old animals. At tumor take (30 days post-implantation), animals were treated i.p three times a week with NSC12 (5.0 mg/kg body wt) or vehicle. Tumors were measured in two dimensions and volume was calculated according to the formula V = (D × d^2^)/2, where D and d are the major and minor perpendicular tumor diameters, respectively. After 17 days, tumors were harvested, embedded in OCT compound, and immediately frozen.

### 4.10. Immunoflurescence Analysis

Control and *ptx3b* MO injected embryos were fixed in 4% paraformaldehyde for 2 h at room temperature. Whole-mount immunofluorescence analysis of cilium axoneme was performed using a mouse anti-acetylated α-tubulin antibody (1:1000, Sigma) followed by Alexa Fluor 488 anti-mouse IgG (Molecular Probes, Eugene, OR, USA).

Tumor cells were seeded on glass coverslips and serum-starved for 48–72 h. Then, cells were fixed in 4% paraformaldehyde, permeabilized with 0.5% Triton X-100, saturated with 3% BSA in PBS, and incubated with an anti-acetylated α-tubulin antibody (1:1000, Sigma) and an anti-γ-tubulin antibody (1:1000, Sigma). Secondary antibodies anti-mouse Alexa Fluor 488 and anti-rabbit Alexa Fluor 594 (Molecular Probes) were used at 1:300 dilutions. Nuclei were counterstained with DAPI.

For tumor samples, 15 µm cryostat sections were air dried, fixed with 4% paraformaldehyde for 15 min, and permeabilized with 0.5% Triton X-100/PBS for 15 min at room temperature (RT). Sections were blocked for 2 h at RT with 1% BSA, 0.1% fish gelatin (Sigma-Aldrich), 0.1% Triton X-100 and 0.05% Tween20 (blocking solution), and incubated for a further 1 h at RT with a M.O.M blocking reagent (Vector Laboratories, Burlingame, CA, USA) to block endogenous mouse IgGs. Then, sections were incubated overnight at 4 °C with anti-acetylated α-tubulin antibody (1:1000 in blocking solution) and for one further hour at RT with anti-mouse Alexa Fluor 488 secondary antibody (1:250) in blocking solution. Nuclei were counterstained with DAPI.

Immunostained cells, embryos, and tissue sections were analyzed using a Zeiss Axiovert 200M epifluorescence microscope equipped with a Plan-Apochromat × 63/1.4 NA oil objective. Cilium length (in µm) was quantified manually using the “segmented line” tool of ImageJ (NIH).

### 4.11. Western Blot Analysis

Five zebrafish embryos were pooled for each experimental point and were sonicated in sample buffer (250 mM Tris-HCl pH 6.8, 8% SDS, 40% glycerol, 200 mM DTT, 0.02% bromophenol blue), while tumor cells were directly harvested in the same buffer. The protein concentration in the lysates was assessed using the Bradford protein assay (Bio-Rad Laboratories, Milano, Italy). Then, lysates were run on a SDS-PAGE gel, transferred onto a PVDF membrane, and immunocomplexes were visualized by chemiluminiscence (Bio-Rad). The following primary antibodies were used: rabbit anti-pFGFR1 (1:1000, Santa Cruz Biotechnology, Dallas, TX, USA), mouse anti-α-tubulin (1:1000, Sigma), rabbit anti-trichoplein (TCHP) (1:700, Bioss, Woburn, UK), mouse anti-β-actin (1:1000, Sigma). The whole blots showing all the bands with molecular weight markers are shown in Figure S5.

## 5. Conclusions

In summary, our study demonstrates the ability of the natural FGF trap *PTX3* to exert a modulatory effect on primary cilium in embryonic development and cancer. Moreover, they set the basis for the design of novel *PTX3*-derived ciliogenic drugs with potential implications for the therapy of FGF-dependent tumors.

## Figures and Tables

**Table 1:** Oligonucleotide primers used for RT-PCR and qPCR analysis.

Gene	Forward	Reverse
*ptx3b*	5′-TTGGCAGACTGAAGACATGG-3′	5′-GGGCAGATAGCGGTTTACGGT-3′
*ptx3b skipping exon*	5′-ACGTACCAGAATGTATGCG-3′	5′-CATGGAGGGTGTTACTTC-3′
*β-actin*	5′-CGAGCAGGAGATGGGAACC-3′	5′-CAACGGAAACGCTCATTGC-3′
*ef-1a*	5′-GGTACTTCTCAGGCTGACTGT-3′	5′-CAGACTTGACCTCAGTGGTTA-3′
*Gli1*	5′-TTCAAGGCCCAATACATGCT-3′	5′-GCGTCTTGAGGTTTTCAAGG-3′
*Gapdh*	5′-CATGGCCTTCCGTGTTCCTAC-3′	5′-TTGCTGTTGAAGTCGCAGGAG-3′

## Supplementary Materials

The following figures are available online as a single pdf file at https://www.mdpi.com/2072-6694/12/7/1756/s1, Figure S1: *ptx3a* and *ptx3b* are zebrafish co-orthologue genes of human *PTX3*, Figure S2: Zebrafish *ptx3a*/*ptx3b* and human *PTX3* synteny, Figure S3: *ptx3b* knockdown causes defects in liver and pancreas positioning in zebrafish embryo, Figure S4: Effect of *PTX3* overexpression on primary cilium length in TRAMP-C2 cells, Figure S5: *hPTX3* overexpression does not affect the percentage of ciliated tumor cells, Figure S6: Effect of NSC12 on the proliferation of TRAMP-C2 cells, Figure S7: Anti-α-tubulin immunoreactivity in TRAMP-C2 tumors, Figure S8: RT-PCR raw data, Figure S9: Western blot raw data.
