# Supplementary material for: Long-Pentraxin 3 Affects Primary Cilium in Zebrafish Embryo and Cancer Cells via the FGF System
Source: Cancers (Basel). 2020 Jul 1;12(7):1756. doi: 10.3390/cancers12071756 (PMC7409334; doi:10.3390/cancers12071756)
Supplement: Supplementary file 1 [file cancers-12-01756-s001.pdf]

cancers

MDPI

Article

Long-pentraxin 3 affects primary cilium in zebrafish embryo and cancer cells *via* the FGF system

Jessica Guerra, Paola Chiodelli, Chiara Tobia, Claudia Gerri and Marco Presta

Supplementary Materials

```
hPTX3       --------------MHLLAILFCALWSA---VLAENSDDYDLMYV-NLDNEIDNG-----     37
zptx3a      ---------MASKSMWTFLQTMCLVASLSMAVSDNYDDDIEVGFGHNYFNEIPDEDQLED     51
zptx3b      MQRETNVHRMYAPGAVLALCLPCFLTGI------VFSYEYGEDYSDSYYNEISNGDRVQS     54
                                  * : .         . :     :  .  *** :

hPTX3       LHPTEDPTPCDCGQEHSEWDKLFIMLENSQMRERMLLQATDDVLRGELQRLREELGRLAE     97
zptx3a      ETPTPSPEPCK-NTAFTKWDKLFTMLENSQMKENMLLQYADDIIKVELQNLRGEMIQFVA     110
zptx3b      E---GTQIPCP-PQDLSRWDKLFIMLEDSQMKQNMLHWQTEDMVKKEMDSIRKDLHKLNN     110
                    **       :.***** ***:***::.**    ::*:::  *:: :* :: ::

hPTX3       SLARPCAPGAPAEARLT--------------------------SALDELLQATRDAGRR     130
zptx3a      QYGGSCASAVESSVRRAGTQTESCLQNALDRAREVSADRYEQHDAVLQQLLAVSVNQAAQ     170
zptx3b      N--KACVQASENTCKCISEQMNRNIKQVMEQLREAADSHQAQNNETLQQLIQLSRHQASR     168
            .    *. .      :                                .*::*:  : . . :

hPTX3       LARMEGAE--------------------AQRPEEAGRALAAVLEELRQTRADLHAVQGWA     170
zptx3a      LDRLEKGCLKSGAFKNIQTRHLMQDVTEIENGGKLEKSLETISSELQSIREQLSLYTRSA     230
zptx3b      LAKLELGAGLGQVAVKSFSVYPKEQEASSADGGKLERALMAATADLQRVHAQLALFQRAT     228
            * ::* .                             :  ::* :   :*:  : :*      :

hPTX3       ARSWLPAGCETAILFPMRSKKIFGSVHPVRPMRLESFSACIWVKATDVLNKTILFSYGTK     230
zptx3a      FADKLPSGCDTGLLFPTRSSKAHAEVSPHGLLRTNAVTICLWVKPTQVFNKTILFSYSTT     290
zptx3b      VNRYLPSGCEMALLFPMRSKHIFAEVTPSMSMSLQTFTLCLWAKVTQALNKTVLFSYGSK     288
                **:**: .:*** **.: ...* *   :  ::.: *:*.* *:.:***:****.:.

hPTX3       RNPYEIQLYLSYQSIVFVVGGEENKLVAEAMVSLGRWTHLCGTWNSEEGLTSLWVNGELA     290
zptx3a      GNAYELQLVLSGQSASFTVDGESHLVEASGAVQEGQWGHLCASWSSQQGLASLWVNGQQA     350
zptx3b      INPQELQLLLSGRSLLLKVGGDTGLVEALGVVTDGQWGHFCATWSSKQGLADLWVNGESV     348
             *  *:** ** :*  : *.*:   : * . *  *:* *:*.:*.*::**:.*****: .

hPTX3       ATTVEMATGHIVPEGGILQIGQEKNGCCV----GGGFDETLAFSGRLTGFNIWDSVLSNE     346
zptx3a      ASSPGVAEGHELRSKGSILLGQEYGRHYKHLSTPDTFDAELAFTGKMTGVNMWDRVLDPK     410
zptx3b      ARVSGVAEGHELPSDGFILLGQEISRSGI----YRDLVSTVVFTGKMTGVNMWDHVLEAE     404
            *    :* ** : . * : :*** .                :   :.*:*::**.*:** **. :

hPTX3       EIRETGG--AESCHIRGNIVGWGVTEIQPHGGAQYVS     381
zptx3a      EISQQARLYGTACGIRGNVVAWGVSDIQPKEGVKLLY     447
zptx3b      RIKEYANQ-DGSCDSRGNVIGWGISEIVPNGGAQYIN     440
            .* : .     :*  ***::.**:::* *: *.: :
```

1: hPTX3	100.00	39.74	41.13
2: zptx3a	39.74	100.00	39.44
3: zPTX3b	41.13	39.44	100.00

Figure S1. *ptx3a* and *ptx3b* are zebrafish co-orthologue genes of human *PTX3*. (**A**) CLUSTAL Omega alignment of the FASTA sequences of hPTX3, zPtx3a, zPtx3b with percentages of identity between sequences. Red box highlights conserved cysteine residues among the proteins while yellow box indicates the conserved "pentraxin signature".

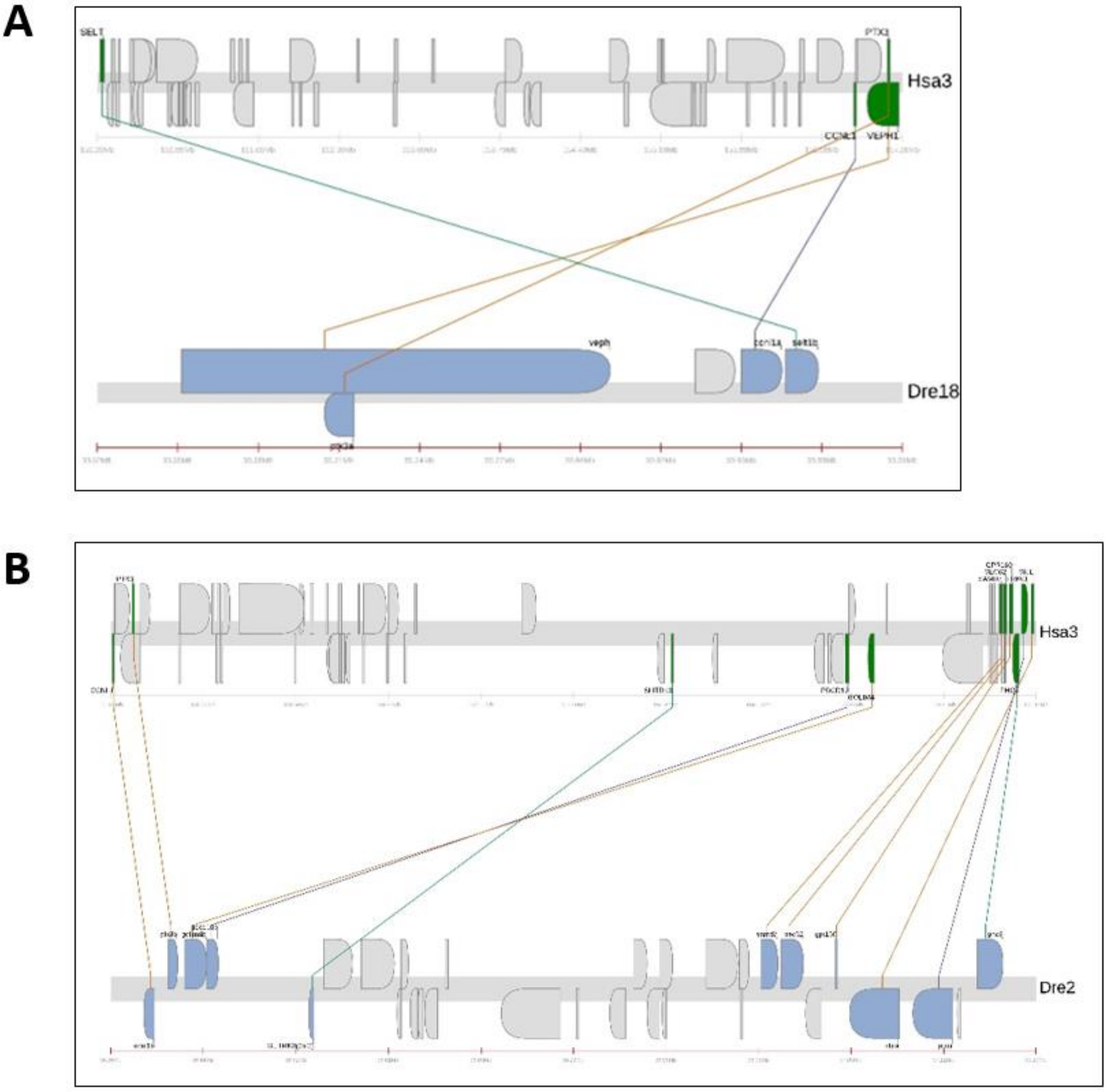

Figure S2. Zebrafish *ptx3a*/*ptx3b* and human *PTX3* synteny. Graphical representation obtained on the Synteny Database Program of the conserved syntenic clusters between *hPTX3* on chromosome 3 (Hsa3) and *zptx3a* on chromosome 18 (Dre18) **(A)** and between *hPTX3* and *zptx3b* on chromosome 2 (Dre2) **(B)**, considering a 50 gene sliding window. Genes are represented as squares; colored squares represent genes conserved in the cluster taken in exam.

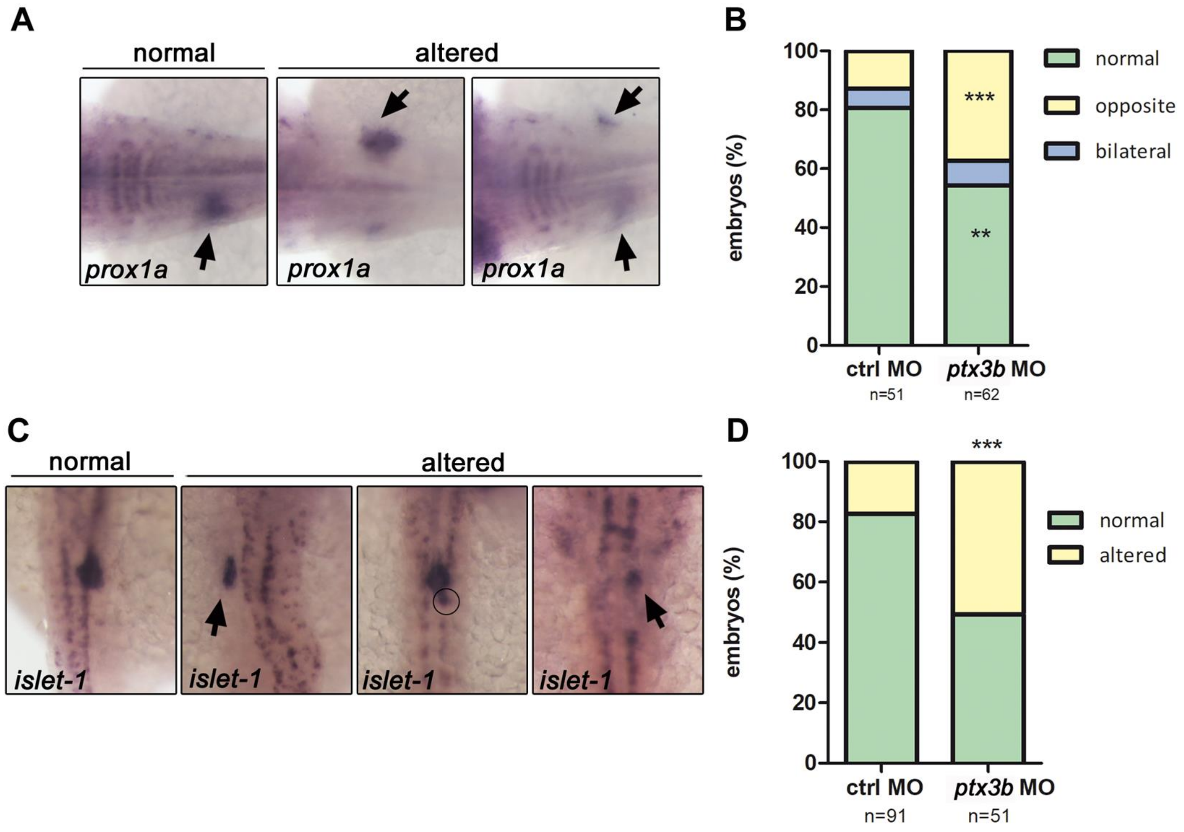

Figure S3. *ptx3b* knockdown causes defects in liver and pancreas primordium positioning in zebrafish embryo. **(A)** Representative WISH pictures at high magnification of the *prox1b*$^{+}$ liver primordium in 48 hpf embryos after injection of ctrl or *ptx3b* MO. Arrows indicate liver primordium. Dorsal, anterior to the left. **(B)** Percentage of embryos with normal, opposite or bilateral liver positioning in ctrl and *ptx3b* MO-injected embryos. Data are from 2 independent experiments, **p<0.01, ***p<0.001, ANOVA. n, total number of analyzed embryos. **(C)** Representative WISH pictures at high magnification of the *islet-1*$^{+}$ dorsal pancreatic bud in 24 hpf embryos after injection of ctrl or *ptx3b* MO. Arrows and circle indicate altered dorsal pancreatic bud localization. Dorsal, anterior to the top. **(D)** Percentage of embryos with normal or altered dorsal pancreatic bud positioning in ctrl and *ptx3b* MO-injected embryos. Data are from 3 independent experiments, ***p<0.001, Fisher's exact test. n, total number of analyzed embryos.

.

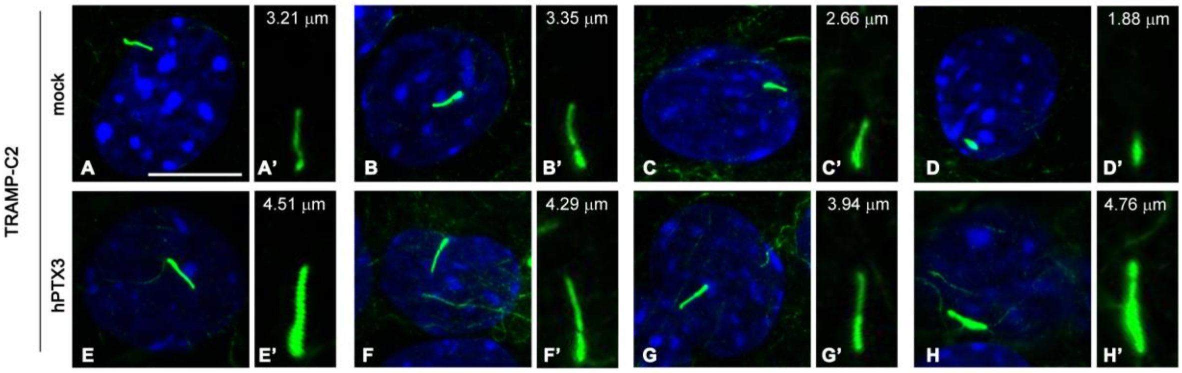

Figure S4. Effect of *PTX3* overexpression on primary cilium length in TRAMP-C2 cells. Representative images of mock (A-D') and *hPTX3*-overexpressing (E-H') TRAMP-C2 cells immunostained with anti-acetylated α-tubulin antibodies (green) to visualize primary cilium axoneme. Nuclei were counterstained with DAPI (blue). For each cell, the length of primary cilium is reported in the adjacent high magnification image. Similar results were obtained for *hPTX3*-overexpressing 5637 and MC17-51 cells (not shown). Scale bar: 10 μm.

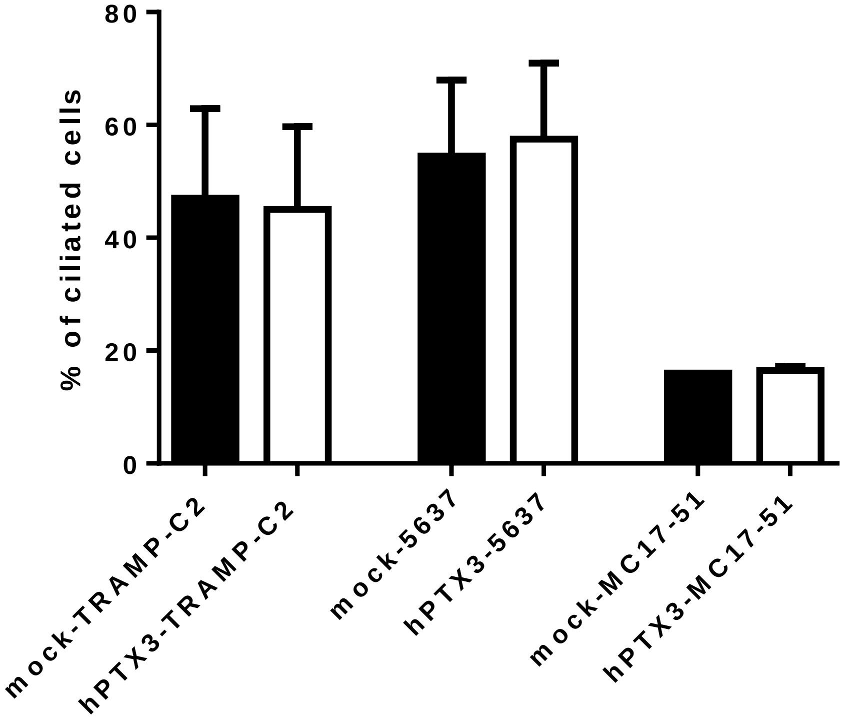

Figure S5. *hPTX3* overexpression does not affect the percentage of ciliated tumor cells. Mock and *hPTX3*-overexpressing tumor cells were stained with anti-acetylated α-tubulin antibodies. Next, the number of ciliated cells were counted (n = 100-200 cells per each cell line).

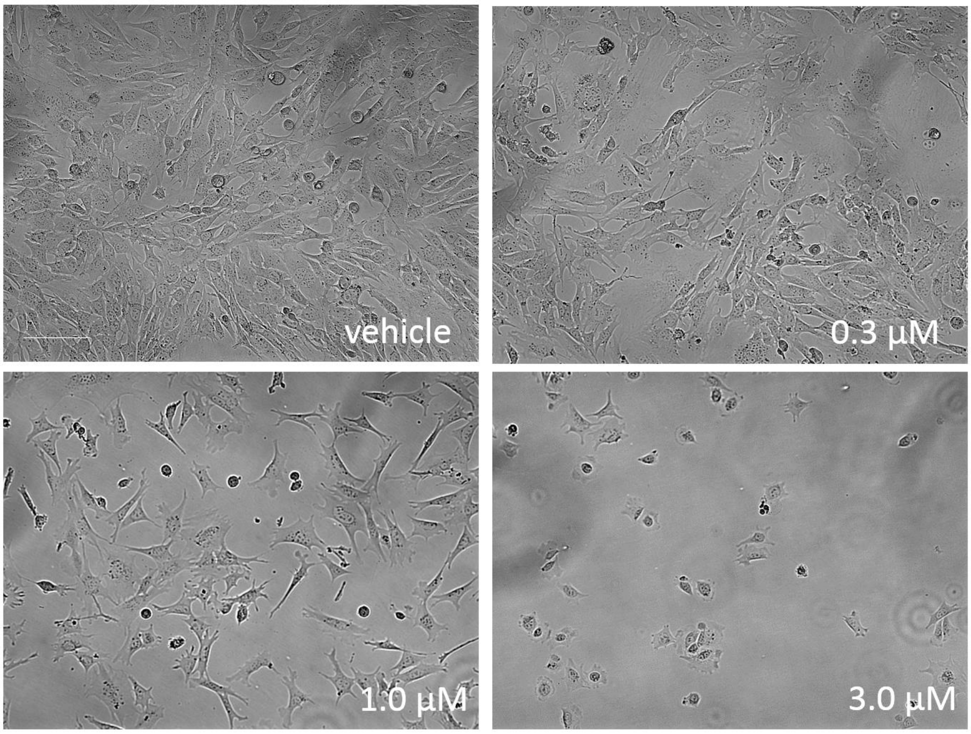

Figure S6. Effect of NSC12 on the proliferation of TRAMP-C2 cells. Serum-starved TRAMP-C2 cells were treated for 48 h with the indicated concentrations of NSC12. At the end of the incubation, cells were photographed under an inverted microscope in bright field at 100x magnification (scale bar: 200 μm). Similar results were obtained for NSC12-treated 5637 and MC17-51 cells (not shown).

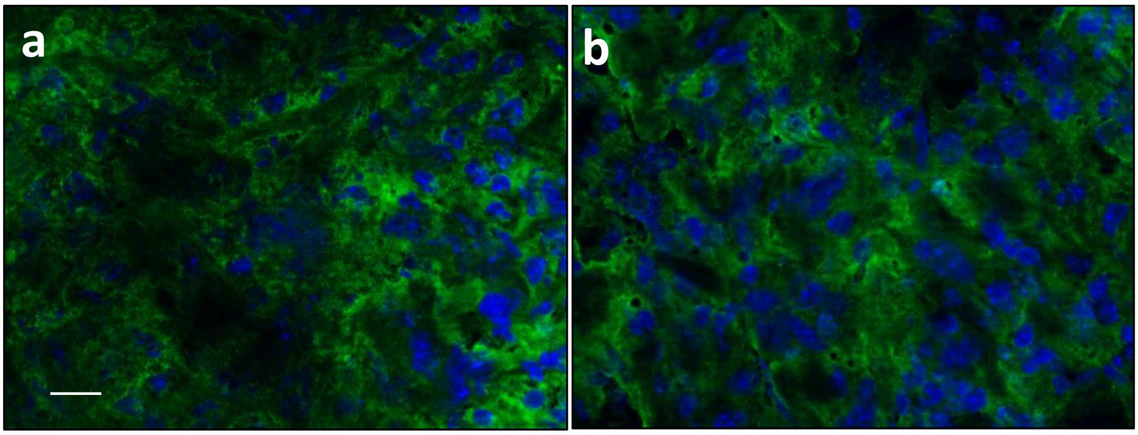

Figure S7. Anti-α-tubulin immunoreactivity in TRAMP-C2 tumors treated with vehicle (a) or NSC12 (b). Nuclei were counterstained with DAPI (blue). No significant difference was observed between control and NSC12-treated grafts. Scale bar: 15 μm.

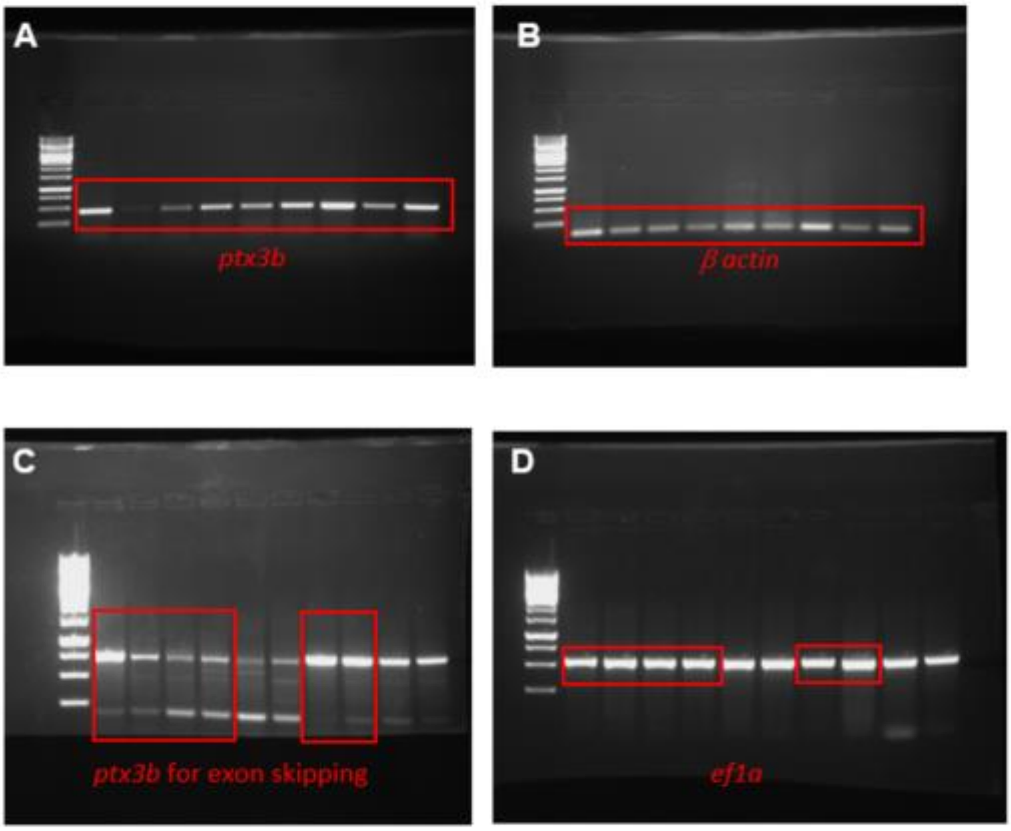

Figure S8. RT-PCR raw data. **(A,B)** Agarose gels of RT-PCR analysis for *ptx3b* and *β-actin*, respectively. Red boxes indicate the lines shown in **Figure 1A**. **(C,D)** Agarose gels of RT-PCR analysis for *ptx3b* and *ef1a*, respectively. Red boxes indicate the lines shown in **Figure 2A**.

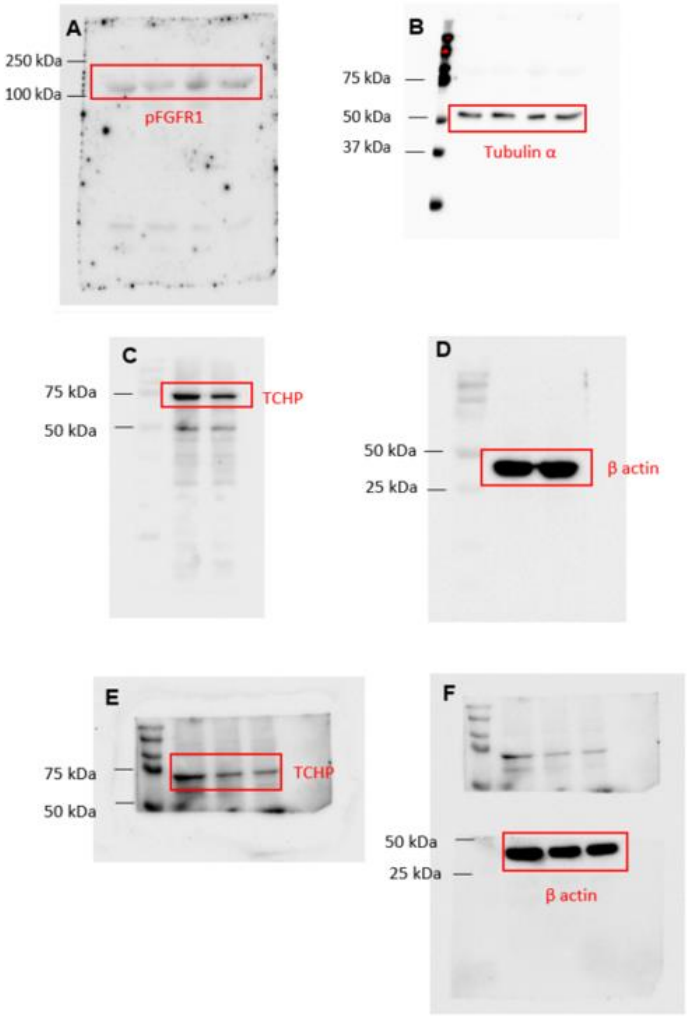

Figure S9. Western Blot raw data. Red boxes in **(A,B)** and **(C,D)** indicate the lines shown in **Figure 4A** and **Figure 7A,** respectively. In both cases, the same membrane was incubated with the indicated Abs and acquired at two different times. **(E, F)** The membrane was cut in two pieces that were incubated with the indicated Abs and acquired separately. Red boxes indicate the lines shown in **Figure 7C**. All the membranes were acquired with a BioRad ChemiDoc Imaging System.

© 2020 by the authors. Licensee MDPI, Basel, Switzerland. This article is an open access article distributed under the terms and conditions of the Creative Commons Attribution (CC BY) license (http://creativecommons.org/licenses/by/4.0/).
